# Overlaps of multiple database retrieval and citation tracking in dementia care research: a methodological study

**DOI:** 10.5195/jmla.2021.1129

**Published:** 2021-04-01

**Authors:** Julian Hirt, Johannes Bergmann, Melanie Karrer

**Affiliations:** 1 julian.hirt@ost.ch, Center for Dementia Care, Institute of Applied Nursing Sciences, FHS St.Gallen, University of Applied Sciences, Department of Health, Rosenbergstrasse 59, 9000 St.Gallen, Switzerland and International Graduate Academy, Institute for Health and Nursing Science, Medical Faculty, Martin Luther University Halle-Wittenberg, Magdeburger Strasse 8, 06112 Halle (Saale), Germany; 2 Johannes-Michael.Bergmann@dzne.de, German Centre for Neurodegenerative Diseases (DZNE), Stockumer Strasse 12, 58453 Witten, Germany and University Witten/Herdecke, Faculty of Health, Department for Nursing Science, Stockumer Strasse 12, 58453 Witten, Germany; 3 melanie.karrer@ost.ch, Center for Dementia Care, Institute of Applied Nursing Sciences, FHS St.Gallen, University of Applied Sciences, Department of Health, Rosenbergstrasse 59, 9000 St.Gallen, Switzerland

**Keywords:** database, literature searching, dementia, implementation science, evidence-based nursing

## Abstract

**Objective:**

We aimed to determine overlaps and optimal combination of multiple database retrieval and citation tracking for evidence synthesis, based on a previously conducted scoping review on facilitators and barriers to implementing nurse-led interventions in dementia care.

**Methods:**

In our 2019 scoping review, we performed a comprehensive literature search in eight databases (CENTRAL, CINAHL, Embase, Emcare, MEDLINE, Ovid Nursing Database, PsycINFO, and Web of Science Core Collection) and used citation tracking. We retrospectively analyzed the coverage and overlap of 10,527 retrieved studies published between 2015 and 2019. To analyze database overlap, we used cross tables and multiple correspondence analysis (MCA).

**Results:**

Of the retrieved studies, 6,944 were duplicates and 3,583 were unique references. Using our search strategies, considerable overlaps can be found in some databases, such as between MEDLINE and Web of Science Core Collection or between CINAHL, Emcare, and PsycINFO. Searching MEDLINE, CINAHL, and Web of Science Core Collection and using citation tracking were necessary to retrieve all included studies of our scoping review.

**Conclusions:**

Our results can contribute to enhancing future search practice related to database selection in dementia care research. However, due to limited generalizability, researchers and librarians should carefully choose databases based on the research question. More research on optimal database retrieval in dementia care research is required for the development of methodological standards.

## INTRODUCTION

High-quality and effective interventions are key components of evidence-based health care [1]. Methods promoting an optimal uptake of research findings into practice are the subject of implementation science [2]. Implementation science systematically and comprehensively analyzes contextual components of the development, piloting, and evaluation of interventions. Considering contextual components such as facilitators and barriers to implementation might help to plan high-quality health interventions and improve effectiveness [3, 4].

Evidence mapping and synthesis methods enable researchers to consider contextual components of implementation, e.g., facilitators and barriers [5]. Such influencing components are frequently reported in process evaluations of interventional studies [4]. Therefore, systematic and ongoing evidence syntheses are necessary to inform researchers and practitioners about the latest evidence on implementation concerns. This evidence should be considered when developing, piloting, or evaluating interventions in dementia care.

For evidence synthesis, electronic database retrieval and the use of supplementary search methods are core components of systematic literature searching as indicated by current methodological guidance and expert consent [6–8]. Databases cover different topics and references, but also show overlaps [9–11]. The use of multiple databases has increased over the last three decades [12, 13]; however, database overlaps might not be transparent to researchers and, therefore, remain unclear or can only be estimated [14–16]. The use or non-use of an electronic health database for systematic literature searching might depend on the search approach (e.g., sensitive or specific), major database topic(s) according to the research question or a component of it (e.g., CINAHL for nursing and midwifery, PEDro for physiotherapy, or national or local databases), intended study and publication type(s) (e.g., CENTRAL for randomized controlled trials and OpenGrey for grey literature), commonness of its use (MEDLINE, Embase, and Cochrane Library), and accessibility due to institutional licenses [11, 13, 17]. The variety of such options and an associated lack of clarity about database coverage and overlaps might challenge the selection process. Nevertheless, the selection and combination of suitable, necessary, and most appropriate electronic databases should be carefully justified, since searching multiple databases is time-consuming [18].

To guide researchers, medical librarians or information specialists in choosing relevant databases, health-related research provides evidence on (1) coverage and overlaps of specific databases or how database usage can be optimally combined for efficient search strategies [19–23], and on (2) optimized search approaches to retrieve specific study designs such as qualitative studies [15, 24, 25], trials [10, 26–28], reviews [29] or studies from specific countries [30,31]. Furthermore, there are clear guidelines on database use, e.g., for conducting Cochrane reviews [32]. Specifically, for dementia care research, Frandsen et al. [33] determined the coverage of PubMed according to eligible references in dementia-related Cochrane reviews. The authors concluded that approximately three out of four references might be covered by searching PubMed. Further research on the use and retrieval of (multiple) databases for evidence synthesis in dementia care research is lacking.

In sum, evidence synthesis requires the use of multiple databases for a systematic literature search [7, 10, 32]. Particularly in dementia care research, it is unclear which combination of databases might be optimal to search as efficiently as possible (i.e., to retrieve most of the eligible references by using a minimum number of databases). Therefore, we aimed to determine the overlaps and optimal combination of multiple database retrieval and citation tracking for evidence synthesis using data from an existing scoping review on a dementia-specific research question [34].

## METHODS

### Scoping review

We conducted a methodological study based on the search strategies and results of a previous scoping review [34]. In our scoping review, we included qualitative, quantitative, and mixed methods studies on facilitators and barriers to implementing nurse-led interventions in dementia care published since 2015. In January 2019, we searched the following eight electronic databases: CENTRAL via Cochrane Library, CINAHL, Embase via Ovid, Emcare, MEDLINE via Ovid, Ovid Nursing Database, PsycINFO via Ovid, and Web of Science Core Collection. Two authors experienced in dementia care research (JH, MK) created the search strategies. Our search strategies contained topical free-text terms and database-specific controlled vocabulary. To ensure the accuracy of the search process, we applied Peer Review of Electronic Search Strategies (PRESS) [35]. The final database-specific search strategies are shown in the supplemental files (Appendix A: Search strategies). Databases were chosen according to the topic of the scoping review. Table 1 displays the characteristics of databases retrieved in our scoping review.

**Table 1:** Characteristics of retrieved databases

Database	Interface	Access	Type	Coverage
CENTRAL	Cochrane Library	Free of charge	Indexed database	Health
CINAHL	EBSCO	Subscription-based	Indexed database	Health, i.e. nursing
Embase	Ovid	Subscription-based	Indexed database	Health, biomedicine, pharmacology
Emcare	Ovid	Subscription-based	Indexed database	Health, i.e. nursing
MEDLINE	Ovid	Subscription-based	Indexed database	Health, biomedicine
Ovid Nursing Database	Ovid	Subscription-based	Indexed database	Nursing
PsycINFO	Ovid	Subscription-based	Indexed database	Health, i.e. psychology
Scopus	Elsevier	Subscription-based	Citations database, indexed database	Health, biomedicine, life sciences, technology, art, social sciences
Web of Science Core Collection	Web of Science	Subscription-based	Citations database, indexed database	Across scientific disciplines

Handsearching, free web searching, and citation tracking of included studies using Scopus supplemented our search approach [7]. For our citation tracking process, we used Scopus, since it covers the largest number of studies in health-related disciplines [34]. We conducted backward citation tracking (to identify cited references) and forward citation tracking (to identify citing references) based on the included studies retrieved by database searching and supplementary search methods (see above). After eligibility screening of the studies retrieved by citation tracking, we identified two relevant studies for our scoping review. Based on these newly identified references, we started another round of backward and forward citation tracking, resulting in no additional eligible studies. Further methodological details of the scoping review (e.g., eligibility criteria, development of the search strategies, and data analysis) are provided elsewhere [34]. We included 26 studies in our scoping review [34].

We imported all references retrieved from electronic database searching and citation tracking in IBM SPSS Statistics 25. These references represented the end search results of our scoping review.

We did not find sufficient methodological details on how authors of previous studies determined overlaps and optimal combination of information sources. Therefore, we inductively developed target-oriented methods for measurement, described here. Within our dataset, rows represented cases (number of references) and columns represented variables (characteristics of references). Our assigned variables included bibliographic data references (e.g., year, title, author[s], and digital objective identifier [DOI]), unique or duplicate retrieval, name of database retrieved, and inclusion in our scoping review or exclusion during title/abstract or full text screening. We sorted references by DOI representing one case per reference in rows with variables assigned in columns, and we manually searched and entered any missing bibliographic data. To calculate the number of duplicates per case and database overlap, we restructured duplicates into variables, thus reducing duplicates to a single case with several databases as variables. In our study, we used the term “duplicates” to indicate the total number of multiple identical references (e.g., five references indexed twice will result in ten duplicates) and “duplicate cases” for the reduction of multiple identical references to one case (e.g., five references indexed twice will result in five duplicate cases). Study data is provided as an SPSS file in our supplementary study material at Open Science Framework (see “Data Availability Statement”).

We analyzed database overlaps (duplicate cases captured by multiple databases) and unique references using cross tables and descriptive statistics. Additionally, we analyzed database similarity using multiple correspondence analysis (MCA) [36]. MCA is a descriptive data analysis technique that simplifies the presentation of complex data by reducing dimensions. This method is used in health sciences to describe similarities between characteristics and to illustrate data based on a Burt table or complete disjunctive table [37–39]. In this way, MCA can graphically represent both row and column characteristics of a complete disjunctive table in the same low-dimensional space [40]. Therefore, we applied MCA to a complete disjunctive table with references in rows and databases in columns.

Deviation of row or column profiles from their respective average profile is a measure of variance in the data. In the context of MCA, this measure of variance is designated as inertia. In summary, MCA calculates the singular value decomposition of a complete disjunctive table, yielding a set of eigenvalues (λs) and corresponding eigenvectors (dimensions). The total inertia is based on the MCA's eigenvalues. The aim is to calculate the best low-dimensional solution (usually two- or three-dimensional) in order to distinguish geometric patterns in the data. Data visualization by MCA usually aims at a low-dimensional (two- to three-dimensional) representation resulting in a loss of information [41]. However, we have chosen this method to provide a concise two-dimensional graphic representation of databases’ overlaps. This so-called MCA map is illustrated as a Cartesian coordinate system. The first dimension (λ1, inertia of first dimension) of the MCA map corresponds to the x-axis and explains a certain amount of the total inertia (given in percent). The second dimension (λ2, inertia of second dimension) corresponds to the y-axis and explains a certain amount of the total inertia (given in percent). For interpretation of the MCA map, a database containing all references would be located at the center (coordinate origin), and a low-frequency database (e.g., a database containing few references) is far away from the center. The distance between two or more databases shows their similarities.

To conduct statistical analyses, we used the statistical software R [42]. We performed MCA analyses with the R package “FactoMineR” using the MCA function [43]. The R-files are provided in our supplementary study material at Open Science Framework (see “Data Availability Statement”).

## RESULTS

### Database coverage and overlaps

Our search in eight electronic databases and citation tracking of included studies yielded 10,527 studies published between 2015 and 2019. Of these, 6,944 were duplicates and 3,583 were unique references. Table 2 displays overall duplicates as well as duplicates included in our scoping review and unique references per database.

**Table 2:** Duplicates and unique references per database for overall and included studies

	Overall (n)		Included (n)	
	Duplicates	Uniques	Duplicates	Uniques
CENTRAL	214	176	5	0
CINAHL	832	220	15	2
Embase	609	227	9	0
Emcare	1065	550	9	0
MEDLINE	1640	280	16	3
Ovid Nursing Database	223	4	5	0
PsycINFO	649	148	11	0
Citation Tracking via Scopus	88	205	6	2
Web of Science Core Collection	1624	1773	15	1
Total	6944	3583	91	8

#### Unique references (n=3,583)

According to Table 2, Web of Science Core Collection provided the highest number of unique references (n=1,773), followed by Emcare (n=550). Ovid Nursing Database offered the lowest number of unique references (n=4). The eight unique references we included in our scoping review were retrieved from MEDLINE (n=3), CINAHL (n=2), citation tracking via Scopus (n=2), and Web of Science Core Collection (n=1).

#### Duplicates (n=6,994)

Most duplicates were indexed in MEDLINE (n=1,640) and Web of Science Core Collection (n=1,624). We retrieved the fewest duplicates from citation tracking via Scopus (n=88). Duplicates included in our scoping review were retrieved from all databases, mostly MEDLINE (n=16) and CINAHL (n=15), and from Web of Science Core Collection (n=15). The included 91 duplicates (Table 2) represent 18 duplicate cases (single references).

Among the retrieved 6,944 duplicates, we identified 1,944 duplicate cases (single references). Cases had between two and nine duplicates (mean=3.6; median=3). We retrieved the most cases from two databases (n=618) and the fewest cases from all databases (n=2). Table 3 shows database overlap of indexed and non-indexed cases among retrieved duplicate cases (n=1,944). For each database searched and citation tracking conducted, indexed (In) and non-indexed (Out) cases are shown in rows and columns. Bold numbers represent the total number of duplicate cases indexed in each database. Cross-tabulated reading provides a detailed overview of database overlap. For example, of 214 duplicate cases indexed in CENTRAL, 94 are also indexed in CINAHL, whereas 120 are not indexed in CINAHL. A second example: of 320 duplicate cases not indexed in Web of Science Core Collection, 216 are retrieved through MEDLINE via Ovid.

**Table 3:** Database overlaps of indexed (In) and non-indexed (Out) cases among retrieved duplicate cases (n=1,944)

		CENTRAL		CINAHL		Embase		Emcare		MEDLINE via Ovid		Ovid Nursing Database		PsycINFO		Citation Tracking via Scopus		Web of Science Core Collection	
		In	Out	In	Out	In	Out	In	Out	In	Out	In	Out	In	Out	In	Out	In	Out
CENTRAL	In	**214**	–	94	120	114	100	99	115	153	61	19	195	62	152	11	203	164	50
	Out	–	**1730**	738	992	495	1235	966	764	1487	243	204	1526	587	1143	77	1653	1460	270
CINAHL	In			**832**	–	329	503	583	249	730	102	172	660	373	459	48	784	742	90
	Out			–	**1112**	280	832	482	630	910	202	51	1061	276	836	40	1072	882	230
Embase	In					**609**	–	393	216	533	76	103	506	235	374	43	566	507	102
	Out					–	**1335**	672	663	1107	228	120	1215	414	921	45	1290	1117	218
Emcare	In							**1065**	–	938	127	139	926	381	684	45	1020	905	160
	Out							–	**879**	702	177	84	795	268	611	43	836	719	160
MEDLINE	In									**1640**	–	212	1428	574	1066	60	1580	1424	216
	Out									–	**304**	11	293	75	229	28	276	200	104
Ovid Nursing Database	In											**223**	–	75	148	14	209	169	54
	Out											–	**1721**	574	1147	74	1647	1455	266
PsycINFO	In													**649**	–	40	609	579	70
	Out													–	**1295**	48	1247	1045	250
Citation Tracking via Scopus	In															**88**	–	74	14
	Out															–	**1856**	1550	306
Web of Science Core Collection	In																	**1624**	–
	Out																	–	320

**Figure 1:**
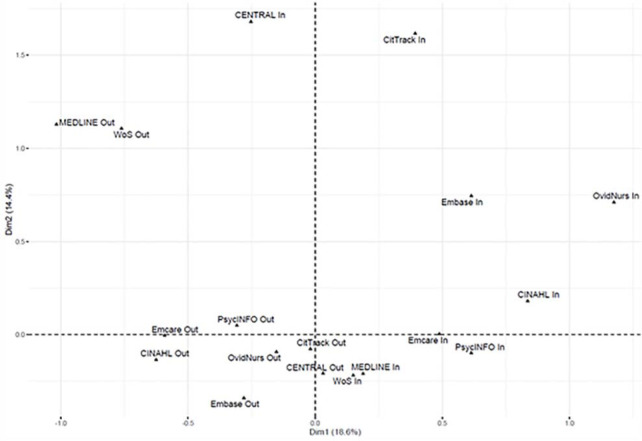
MCA map representing relations between databases indicated by indexed (In) and non-indexed (Out) cases

The MCA map (Figure 1) illustrates the similarity of databases representing data shown in Table 3 and shows two important facts: first, the number of studies that a database contains or does not contain (indicated by the databases’ distances from the center of the MCA and labeled as category “In” (indexed) or “Out” (non-indexed) for each database); second, the similarity of databases (indicated by the distances between different databases). In the MCA map, if we focus on the “In” category, or those that indicate the included references from each database, a database containing more included references is located near the center, and a low-frequency database (i.e., a database containing few included references) is far away from the center. For example, “CENTRAL In,” “CitTrack In,” and “OvidNurs In” contain smaller numbers of references and, therefore, are located far away from the center, while “MEDLINE In” and “WoS In” (Web of Science Core Collection) contain larger numbers of references and are located close to the center. Databases located close to each other are defined as “similar,” and databases distant from each other are defined as “dissimilar.” The most similar databases are MEDLINE and Web of Science Core Collection, with 1,424 of 1,640 (87%) references in MEDLINE that are also indexed in Web of Science Core Collection (Table 3).

CT = Citation Tracking; WoS = Web of Science Core Collection.

### Optimal database combination

Table 4 displays Indexing (In) and Non-indexing (Out) of unique and duplicate cases within included studies [34]. Searching MEDLINE (n=18), CINAHL (n=17), Web of Science Core Collection (n=16), and using citation tracking (n=17) yielded the most included cases. The sample comprised eight unique and 18 duplicate cases. Duplicate cases are indexed in two to eight databases.

**Table 4:** Indexing (In) and non-indexing (Out) of unique and duplicate cases within included studies in our scoping review

Case	Number of duplicate cases	CENTRAL	CINAHL	Embase	Emcare	MEDLINE via Ovid	Ovid Nursing Database	PsycINFO	Citation Tracking via Scopus	Web of Science Core Collection	Minimum necessary database(s)
1	NA	Out	Out	Out	Out	Out	Out	Out	Out	In	WoS
2	NA	Out	Out	Out	Out	In	Out	Out	Out	Out	MEDLINE
3	NA	Out	Out	Out	Out	Out	Out	Out	In	Out	CT
4	NA	Out	Out	Out	Out	In	Out	Out	Out	Out	MEDLINE
5	NA	Out	Out	Out	Out	In	Out	Out	Out	Out	MEDLINE
6	NA	Out	In	Out	Out	Out	Out	Out	Out	Out	CINAHL
7	NA	Out	In	Out	Out	Out	Out	Out	Out	Out	CINAHL
8	NA	Out	Out	Out	Out	Out	Out	Out	In	Out	CT
9	8	In	In	In	In	In	Out	In	In	In	MEDLINE or CINAHL or WoS or CT
10	7	Out	In	In	Out	In	In	In	In	In	MEDLINE or CINAHL or WoS or CT
11	7	Out	In	In	Out	In	In	In	In	In	MEDLINE or CINAHL or WoS or CT
12	7	In	In	In	In	In	In	Out	Out	In	MEDLINE or CINAHL or WoS
13	7	In	In	In	Out	In	In	In	Out	In	MEDLINE or CINAHL or WoS
14	7	In	In	In	In	In	Out	In	Out	In	MEDLINE or CINAHL or WoS
15	6	In	In	Out	Out	In	In	Out	In	In	MEDLINE or CINAHL or WoS or CT
16	6	Out	In	In	In	In	Out	In	Out	In	MEDLINE or CINAHL or WoS
17	6	Out	In	In	In	In	Out	In	Out	In	MEDLINE or CINAHL or WoS
18	5	Out	In	Out	In	In	Out	Out	In	In	MEDLINE or CINAHL or WoS or CT
19	5	Out	In	In	Out	In	Out	In	Out	In	MEDLINE or CINAHL or WoS
20	5	Out	In	Out	In	In	Out	In	Out	In	MEDLINE or CINAHL or WoS
21	4	Out	In	Out	In	In	Out	Out	Out	In	MEDLINE or CINAHL or WoS
22	3	Out	In	Out	Out	Out	Out	Out	In	In	CINAHL or WoS or CT
23	2	Out	Out	Out	Out	In	Out	In	Out	Out	MEDLINE
24	2	Out	Out	Out	Out	In	Out	In	Out	Out	MEDLINE
25	2	Out	In	Out	In	Out	Out	Out	Out	Out	CINAHL
26	2	Out	Out	Out	Out	In	Out	Out	Out	In	MEDLINE or WoS
Sum (In)	NA	5	17	9	9	18	5	11	17	16	Optimal database combination: CINAHL or MEDLINE or WoS or CT

CT = Citation Tracking; WoS = Web of Science Core Collection

Table 1 has already shown that it was necessary at a minimum to search MEDLINE, CINAHL, and Web of Science Core Collection and to use citation tracking to achieve the final study sample of our scoping review, since these databases and citation tracking yielded unique cases (n=8). As illustrated in Table 4, it was required at a maximum to search MEDLINE, CINAHL, and Web of Science Core Collection and to use citation tracking to identify all included studies of our final sample. This corresponds to an optimal database combination. One case each is solely (1) indexed in Web of Science Core Collection or (2) retrieved using CINAHL, Web of Science Core Collection or citation tracking or (3) using MEDLINE or Web of Science Core Collection. Three cases are solely indexed in CINAHL, and two cases were identified by means of citation tracking. Five cases are indexed in either MEDLINE or CINAHL or Web of Science Core Collection or were retrieved through citation tracking. Another five cases are solely indexed in MEDLINE. Eight cases are indexed either in MEDLINE or CINAHL or Web of Science Core Collection.

## DISCUSSION

Based on our study, several conclusions are possible.

First, we found considerable overlap in some databases using our search strategies (e.g., MEDLINE and Web of Science Core Collection, or CINAHL, PsycINFO, and Emcare). MEDLINE and Web of Science Core Collection contained most of the studies retrieved by our search. However, even though MEDLINE and Web of Science Core Collection showed a high amount of overlap, the use of both databases was necessary in our scoping review since they provide unique references indexed in either one or the other database. This underlines the importance of using MEDLINE and Web of Science Core Collection in dementia-related evidence synthesis [33].

The results of Emcare, CINAHL, and PsycINFO were quite similar, with slight differences. All three databases are balanced in the proportion of references included and not included. These three databases are specific to nursing and dementia-associated research fields, such as psychology and psychiatry. Furthermore, a study that compared search strategies showed that CINAHL, especially, provides differentiated subject headings to retrieve qualitative studies in dementia [44]. This might underline the importance of using CINAHL for dementia-specific search strategies; however, since PsycINFO also seems to be highly relevant in dementia care research [44], this indicates the need for further investigation into the optimal use and potential benefit of CINAHL and PsycINFO for evidence synthesis.

Second, searching CENTRAL and Ovid Nursing Database did not result in many references, whereas many references not indexed in these databases are covered by searching MEDLINE or Web of Science Core Collection. However, using them might be an option if other databases are not available or if, as in the case of CENTRAL, a specific search for intervention studies is intended.

Third, based on our scoping review, this study shows that searching CINAHL, MEDLINE, and Web of Science Core Collection plus citation tracking were necessary to retrieve all included studies of our scoping review [34]. Thus, the initial use of eight databases could have been limited to three databases (CINAHL, MEDLINE, and Web of Science Core Collection) and citation tracking. By limiting the number of databases, considerable effort could have been avoided (e.g., adapting strategies to search CENTRAL, Embase, Ovid Nursing Database, and PsycINFO and screening the approximately 4,000 additional studies retrieved by searching these databases [18]). Although the results cannot be generalized due to the unique nature of our study, researchers conducting evidence syntheses in the field of dementia care could use our findings as a guide for selecting databases to potentially save time.

Fourth, our study underlines the need to complement database searching with backward and forward citation tracking to retrieve all studies in our final sample. Other studies have already shown the benefit of using citation tracking [7, 29, 45]; however, based on our study, it is not possible to draw conclusions about the benefit of further supplementary search methods recommended by current methodological guidance such as handsearching or consultation of experts [6]. This should be considered in future methodological research related to study retrieval in dementia care.

Furthermore, the benefit of a rather new methodological concept called co-citations should be investigated. Like citation tracking, the aim of this method is to identify related articles based on citation relationships. However, the starting point is a cited and a citing reference of an article (for example, a cited and a citing reference of an eligible article in a systematic review). Co-citation retrieval identifies the citing references of the cited reference and the cited references of a citing reference [46]; thus, the exploration of these citation relationships might lead to further eligible studies. Preliminary methodological studies and guidance suggest that co-citations might be more effective than traditional backward and forward citation tracking [45, 47, 48]. However, a comprehensive and systematic investigation of co-citations’ benefit is lacking [49].

Fifth, our study was very time-consuming and required substantial resources, particularly related to data processing and management (e.g., manual searching of missing bibliographic data and restructuring duplicates to reduce them to a single case with several databases as variables). Since we did not find sufficient methodological details on how authors of previous reviews determined overlap and the optimal combination of information sources, we inductively developed the target-oriented methods described above. For the scientific and librarian communities to replicate, confirm, and promote these methods, authors of future studies on database overlap and optimal database combination should describe their methods for data processing and management in detail. This might contribute to developing methodological standards, allowing comparable studies to be conducted in a time-saving manner.

Sixth, future methodological research on database retrieval and overlap (e.g., as part of systematic reviews and overviews of reviews) is needed to confirm our findings. To wisely choose databases for efficient evidence synthesis methods, more certainty on optimal database retrieval in dementia care research would be helpful. Since we did not aim to determine whether study conclusions would have been changed if single or multiple references had not been included in our review, this should be considered in future research [9, 50]. This seems necessary to understand which database combination might be optimal to identify relevant studies and to avoid biased study findings and conclusions.

Finally, our results can contribute to enhancing future search practice in dementia care research. Due to limited generalizability, researchers and librarians should carefully choose databases based on the research question and the intended search principle at hand (e.g., a sensitive or specific search principle). Our results should not be seen as a “free pass” to limit the search to CINAHL, MEDLINE, Web of science Core Collection, and to using backward and forward citation tracking. However, based on our study, these information sources seem to be essential to retrieve core studies in dementia care and must therefore not be neglected by searchers intending a comprehensive literature search.

## Data Availability

Supplementary study material contains data associated with this article and is available as SPSS-file (Supplementary A) and R-files (Supplementary B) in the Open Science Framework at https://osf.io/8qve9/ (DOI: 10.17605/OSF.IO/8QVE9).

## SUPPLEMENTAL FILES

**Appendix A:** Database-specific search strategies
