# Supplementary material for: Overlaps of multiple database retrieval and citation tracking in dementia care research: a methodological study
Source: J Med Libr Assoc. 2021 Apr 1;109(2):275–85. doi: 10.5195/jmla.2021.1129 (PMC8270360; doi:10.5195/jmla.2021.1129)
Supplement: Supplementary file 1 — Appendix A: Database-specific search strategies [file jmla-109-2-275-s01.docx]

**Supplementary file: Database-specific search strategies**

[CINAHL 2](#_Toc535691793)

[MEDLINE via Ovid 3](#_Toc535691794)

[Emcare 3](#_Toc535691795)

[PsycINFO via Ovid 4](#_Toc535691796)

[Embase via Ovid 4](#_Toc535691797)

[CENTRAL via Cochrane Library 5](#_Toc535691798)

[Web of Science Core Collection 5](#_Toc535691799)

[Ovid Nursing Database 6](#_Toc535691800)

# CINAHL

Date: 19.01.2019

| # | Enter | Hits |
| --- | --- | --- |
| 1 | (MH DEMENTIA+ OR TI dement* OR AB dement* OR TI alzheimer* OR AB Alzheimer*) | 76808 |
| 2 | (MH Program Implementation OR MH diffusion of innovation OR MH Knowledge Management+ OR TI "action research" OR TI "healthcare innovation" OR TI "bench to bedside" OR TI "barriers and facilitators" OR TI "facilitators and barriers" OR TI "barriers and enablers" OR TI "enablers and barriers" OR TI "know-do gap" OR TI "integrated knowledge" OR TI "integrating knowledge" OR TI "knowledge to action" OR TI "linkage and exchange" OR TI "organi?ational innovation" OR TI "technology transfer" OR TI "research into practice" OR TI implement* OR TI disseminat* OR TI "Information Distribution" OR TI "Innovation Diffusion" OR TI sustainability OR TI knowledge adoption OR AB "action research" OR AB "healthcare innovation" OR AB "bench to bedside" OR AB "barriers and facilitators" OR AB "facilitators and barriers" OR AB "barriers and enablers" OR AB "enablers and barriers" OR AB "know-do gap" OR AB "integrated knowledge" OR AB "integrating knowledge" OR AB "knowledge to action" OR AB "linkage and exchange" OR AB "organi?ational innovation" OR AB "technology transfer" OR AB "research into practice" OR AB implement* OR AB disseminat* OR AB "Information Distribution" OR AB "Innovation Diffusion" OR AB sustainability OR AB knowledge adoption OR TI (translational N (medicine or science or research or process)) OR TI (information N3 dissemination) OR TI (knowledge N (brokering or communication)) OR TI (knowledge N (cycle or development or application)) OR TI (knowledge N (diffusion or exchange)) OR TI (knowledge N (mobili*ation or synthesis)) OR TI (knowledge N2 (translation or transformation)) OR TI (knowledge N (update or utili*ation)) OR TI (research N2 integration) OR TI (research N2 utili?ation) OR TI (research N apply) OR TI "know do gap" OR TI ((transfer* OR transmission) N2 knowledge) OR TI (circulat* N2 knowledge) OR TI (change or changing) N (behavio?r or practice) AB (translational N (medicine or science or research or process)) OR AB (information N3 dissemination) OR AB (knowledge N (brokering or communication)) OR AB (knowledge N (cycle or development or application)) OR AB (knowledge N (diffusion or exchange)) OR AB (knowledge N (mobili*ation or synthesis)) OR AB (knowledge N2 (translation or transformation)) OR AB (knowledge N (update or utili*ation)) OR AB (research N2 integration) OR AB (research N2 utili?ation) OR AB (research N apply) OR AB "know do gap" OR AB ((transfer* OR transmission) N2 knowledge) OR AB (circulat* N2 knowledge) OR AB (change or changing) N (behavio?r or practice)) | 199266 |
| 3 | #1 AND #2 | 2354 |
| 4 | Limiters - Published Date: 20150101-20191231 | 1061 |

# MEDLINE via Ovid

Date: 19.01.2019

| # | Enter | Hits |
| --- | --- | --- |
| 1 | (exp DEMENTIA/ OR dement*.ti,ab. OR alzheimer*.ti,ab.) | 229794 |
| 2 | ("Health Plan Implementation"/ OR "information dissemination"/ OR exp "diffusion of innovation"/ OR "action research".ti,ab. OR "healthcare innovation".ti,ab. OR "bench to bedside".ti,ab. OR "barriers and facilitators".ti,ab. OR "facilitators and barriers".ti,ab. OR "barriers and enablers".ti,ab. OR "enablers and barriers".ti,ab. OR (translational adj (medicine or science or research or process)).ti,ab. OR (information adj3 dissemination).ti,ab. OR knowledge adoption.ti,ab. OR (knowledge adj (brokering or communication)).ti,ab. OR (knowledge adj (cycle or development or application)).ti,ab. OR (knowledge adj (diffusion or exchange)).ti,ab. OR (knowledge adj (mobili*ation or synthesis)).ti,ab. OR (knowledge adj2 (translation or transformation)).ti,ab. OR (knowledge adj (update or utili*ation)).ti,ab. OR "know do gap".ti,ab. OR "know-do gap".ti,ab. OR "integrated knowledge".ti,ab. OR "integrating knowledge".ti,ab. OR "Knowledge Management"/ OR "knowledge to action".ti,ab. OR "linkage and exchange".ti,ab. OR "organi?ational innovation".ti,ab. OR "technology transfer".ti,ab. OR "research into practice".ti,ab. OR (research adj2 integration).ti,ab. OR (research adj2 utili?ation).ti,ab. OR (research adj apply).ti,ab. OR implement*.ti,ab. OR disseminat*.ti,ab. OR "Information Distribution".ti,ab. OR "Innovation Diffusion".ti,ab. OR ((transfer* OR transmission) adj2 knowledge).ti,ab. OR (circulat* adj2 knowledge).ti,ab. OR sustainability.ti,ab. OR (change or changing) adj (behavio?r or practice).ti,ab.) | 593697 |
| 3 | #1 AND #2 | 4509 |
| 4 | limit 3 to yr="2015 -Current" | 1936 |

# Emcare

Date: 19.01.2019

| # | Enter | Hits |
| --- | --- | --- |
| 1 | (exp DEMENTIA/ OR dement*.ti,ab. OR alzheimer*.ti,ab.) | 97704 |
| 2 | ("information dissemination"/ OR exp "diffusion of innovation"/ OR "action research".ti,ab. OR "healthcare innovation".ti,ab. OR "bench to bedside".ti,ab. OR "barriers and facilitators".ti,ab. OR "facilitators and barriers".ti,ab. OR "barriers and enablers".ti,ab. OR "enablers and barriers".ti,ab. OR (translational ADJ (medicine or science or research or process)).ti,ab. OR (information ADJ3 dissemination).ti,ab. OR knowledge adoption.ti,ab. OR (knowledge ADJ (brokering or communication)).ti,ab. OR (knowledge ADJ (cycle or development or application)).ti,ab. OR (knowledge ADJ (diffusion or exchange)).ti,ab. OR (knowledge ADJ (mobili*ation or synthesis)).ti,ab. OR (knowledge ADJ2 (translation or transformation)).ti,ab. OR (knowledge ADJ (update or utili*ation)).ti,ab. OR "know do gap".ti,ab. OR "know-do gap".ti,ab. OR "integrated knowledge".ti,ab. OR "integrating knowledge".ti,ab. OR "Knowledge Management"/ OR "knowledge to action".ti,ab. OR "linkage and exchange".ti,ab. OR "organi?ational innovation".ti,ab. OR "technology transfer".ti,ab. OR "research into practice".ti,ab. OR (research ADJ2 integration).ti,ab. OR (research ADJ2 utili?ation).ti,ab. OR (research ADJ apply).ti,ab. OR implement*.ti,ab. OR disseminat*.ti,ab. OR "Information Distribution".ti,ab. OR "Innovation Diffusion".ti,ab. OR ((transfer* OR transmission) ADJ2 knowledge).ti,ab. OR (circulat* ADJ2 knowledge).ti,ab. OR sustainability.ti,ab. OR (change or changing) ADJ (behavio?r or practice).ti,ab.) | 402697 |
| 3 | #1 AND #2 | 4857 |
| 4 | limit 3 to yr="2015 -Current" | 1717 |

# PsycINFO via Ovid

Date: 19.01.2019

| # | Enter | Hits |
| --- | --- | --- |
| 1 | (exp DEMENTIA/ OR "Alzheimer's Disease"/ OR dement*.ti,ab. OR alzheimer*.ti,ab.) | 95258 |
| 2 | ("information dissemination"/ OR "action research".ti,ab. OR "healthcare innovation".ti,ab. OR "bench to bedside".ti,ab. OR "barriers and facilitators".ti,ab. OR "facilitators and barriers".ti,ab. OR "barriers and enablers".ti,ab. OR "enablers and barriers".ti,ab. OR (translational adj (medicine or science or research or process)).ti,ab. OR (information adj3 dissemination).ti,ab. OR knowledge adoption.ti,ab. OR (knowledge adj (brokering or communication)).ti,ab. OR (knowledge adj (cycle or development or application)).ti,ab. OR (knowledge adj (diffusion or exchange)).ti,ab. OR (knowledge adj (mobili*ation or synthesis)).ti,ab. OR (knowledge adj2 (translation or transformation)).ti,ab. OR (knowledge adj (update or utili*ation)).ti,ab. OR "know do gap".ti,ab. OR "know-do gap".ti,ab. OR "integrated knowledge".ti,ab. OR "integrating knowledge".ti,ab. OR "Knowledge Management"/ OR "knowledge to action".ti,ab. OR "linkage and exchange".ti,ab. OR "organi?ational innovation".ti,ab. OR "technology transfer".ti,ab. OR "research into practice".ti,ab. OR (research adj2 integration).ti,ab. OR (research adj2 utili?ation).ti,ab. OR (research adj apply).ti,ab. OR implement*.ti,ab. OR disseminat*.ti,ab. OR "Information Distribution".ti,ab. OR "Innovation Diffusion".ti,ab. OR ((transfer* OR transmission) adj2 knowledge).ti,ab. OR (circulat* adj2 knowledge).ti,ab. OR sustainability.ti,ab. OR (change or changing) adj (behavio?r or practice).ti,ab.) | 199769 |
| 3 | #1 AND #2 | 2292 |
| 4 | limit 3 to yr="2015 -Current" | 799 |

# Embase via Ovid

Date: 19.01.2019

| # | Enter | Hits |
| --- | --- | --- |
| 1 | (exp DEMENTIA/ OR dement*.ti,ab. OR alzheimer*.ti,ab.) | 38037 |
| 2 | ("information dissemination"/ OR "action research".ti,ab. OR "healthcare innovation".ti,ab. OR "bench to bedside".ti,ab. OR "barriers and facilitators".ti,ab. OR "facilitators and barriers".ti,ab. OR "barriers and enablers".ti,ab. OR "enablers and barriers".ti,ab. OR (translational adj (medicine or science or research or process)).ti,ab. OR (information adj3 dissemination).ti,ab. OR knowledge adoption.ti,ab. OR (knowledge adj (brokering or communication)).ti,ab. OR (knowledge adj (cycle or development or application)).ti,ab. OR (knowledge adj (diffusion or exchange)).ti,ab. OR (knowledge adj (mobili*ation or synthesis)).ti,ab. OR (knowledge adj2 (translation or transformation)).ti,ab. OR (knowledge adj (update or utili*ation)).ti,ab. OR "know do gap".ti,ab. OR "know-do gap".ti,ab. OR "integrated knowledge".ti,ab. OR "integrating knowledge".ti,ab. OR "Knowledge Management"/ OR "knowledge to action".ti,ab. OR "linkage and exchange".ti,ab. OR "organi?ational innovation".ti,ab. OR "technology transfer".ti,ab. OR "research into practice".ti,ab. OR (research adj2 integration).ti,ab. OR (research adj2 utili?ation).ti,ab. OR (research adj apply).ti,ab. OR implement*.ti,ab. OR disseminat*.ti,ab. OR "Information Distribution".ti,ab. OR "Innovation Diffusion".ti,ab. OR ((transfer* OR transmission) adj2 knowledge).ti,ab. OR (circulat* adj2 knowledge).ti,ab. OR sustainability.ti,ab. OR (change or changing) adj (behavio?r or practice).ti,ab.) | 743643 |
| 3 | #1 AND #2 | 1843 |
| 4 | limit 3 to yr="2015 -Current" | 858 |

# CENTRAL via Cochrane Library

Date: 19.01.2019

| # | Enter | Hits |
| --- | --- | --- |
| 1 | (mh DEMENTIA OR dement*:ti,ab,kw OR alzheimer*:ti,ab,kw) | 14794 |
| 2 | (mh "Health Plan Implementation" OR mh "information dissemination" OR mh "diffusion of innovation" OR "action research":ti,ab,kw OR "healthcare innovation":ti,ab,kw OR "bench to bedside":ti,ab,kw OR "barriers and facilitators":ti,ab,kw OR "facilitators and barriers":ti,ab,kw OR "barriers and enablers":ti,ab,kw OR "enablers and barriers":ti,ab,kw OR (translational NEAR (medicine or science or research or process)):ti,ab,kw OR (information NEAR dissemination):ti,ab,kw OR knowledge adoption:ti,ab,kw OR (knowledge NEAR (brokering or communication)):ti,ab,kw OR (knowledge NEAR (cycle or development or application)):ti,ab,kw OR (knowledge NEAR (diffusion or exchange)):ti,ab,kw OR (knowledge NEAR (mobili*ation or synthesis)):ti,ab,kw OR (knowledge NEAR (translation or transformation)):ti,ab,kw OR (knowledge NEAR (update or utili*ation)):ti,ab,kw OR "know do gap":ti,ab,kw OR "know-do gap":ti,ab,kw OR "integrated knowledge":ti,ab,kw OR "integrating knowledge":ti,ab,kw OR mh "Knowledge Management" OR "knowledge to action":ti,ab,kw OR "linkage and exchange":ti,ab,kw OR "organi?ational innovation":ti,ab,kw OR "technology transfer":ti,ab,kw OR "research into practice":ti,ab,kw OR (research NEAR integration):ti,ab,kw OR (research NEAR utili?ation):ti,ab,kw OR (research NEAR apply):ti,ab,kw OR implement*:ti,ab,kw OR disseminat*:ti,ab,kw OR "Information Distribution":ti,ab,kw OR "Innovation Diffusion":ti,ab,kw OR ((transfer* OR transmission) NEAR knowledge):ti,ab,kw OR (circulat* NEAR knowledge):ti,ab,kw OR sustainability:ti,ab,kw OR (change or changing) NEAR (behavio?r or practice):ti,ab,kw) | 44388 |
| 3 | #1 AND #2 | 1020 |
| 4 | #3 with Publication Year from 2015 to 2019 in Trials | 489 |

# Web of Science Core Collection

Date: 19.01.2019

| # | Enter | Hits |
| --- | --- | --- |
| 1 | TS=(dement* OR alzheimer*) | 301765 |
| 2 | TS=("information dissemination" OR "diffusion of information" OR "action research" OR "healthcare innovation" OR "bench to bedside" OR "barriers and facilitators" OR "facilitators and barriers" OR "barriers and enablers" OR "enablers and barriers" OR (translational NEAR (medicine or science or research or process)) OR (information NEAR dissemination) OR knowledge adoption OR (knowledge NEAR (brokering or communication)) OR (knowledge NEAR (cycle or development or application)) OR (knowledge NEAR (diffusion or exchange)) OR (knowledge NEAR (mobili*ation or synthesis)) OR (knowledge NEAR (translation or transformation)) OR (knowledge NEAR (update or utili*ation)) OR "know do gap" OR "know-do gap" OR "integrated knowledge" OR "integrating knowledge" OR "Knowledge Management"/ OR "knowledge to action" OR "linkage and exchange" OR "organi?ational innovation" OR "technology transfer" OR "research into practice" OR (research NEAR integration) OR (research NEAR utili?ation) OR (research NEAR apply) OR implement* OR disseminat* OR "Information Distribution" OR "Innovation Diffusion" OR ((transfer* OR transmission) NEAR knowledge) OR (circulat* NEAR knowledge) OR sustainability OR (change or changing) NEAR (behavio?r or practice)) | 2152090 |
| 3 | #1 AND #2 | 7812 |
| 4 | Indexes=SCI-EXPANDED, SSCI, A&HCI, CPCI-S, CPCI-SSH, ESCI Timespan=2015-2019 | 3406 |

# Ovid Nursing Database

Date: 19.01.2019

| # | Enter | Hits |
| --- | --- | --- |
| 1 | (exp DEMENTIA/ OR dement*.ti,ab. OR alzheimer*.ti,ab.) | 11790 |
| 2 | ("diffusion of innovation"/ OR "action research".ti,ab. OR "healthcare innovation".ti,ab. OR "bench to bedside".ti,ab. OR "barriers and facilitators".ti,ab. OR "facilitators and barriers".ti,ab. OR "barriers and enablers".ti,ab. OR "enablers and barriers".ti,ab. OR (translational adj (medicine or science or research or process)).ti,ab. OR (information adj3 dissemination).ti,ab. OR knowledge adoption.ti,ab. OR (knowledge adj (brokering or communication)).ti,ab. OR (knowledge adj (cycle or development or application)).ti,ab. OR (knowledge adj (diffusion or exchange)).ti,ab. OR (knowledge adj (mobili*ation or synthesis)).ti,ab. OR (knowledge adj2 (translation or transformation)).ti,ab. OR (knowledge adj (update or utili*ation)).ti,ab. OR "know do gap".ti,ab. OR "know-do gap".ti,ab. OR "integrated knowledge".ti,ab. OR "integrating knowledge".ti,ab. OR "Knowledge Management"/ OR "knowledge to action".ti,ab. OR "linkage and exchange".ti,ab. OR "organi?ational innovation".ti,ab. OR "technology transfer".ti,ab. OR "research into practice".ti,ab. OR (research adj2 integration).ti,ab. OR (research adj2 utili?ation).ti,ab. OR (research adj apply).ti,ab. OR implement*.ti,ab. OR disseminat*.ti,ab. OR "Information Distribution".ti,ab. OR "Innovation Diffusion".ti,ab. OR ((transfer* OR transmission) adj2 knowledge).ti,ab. OR (circulat* adj2 knowledge).ti,ab. OR sustainability.ti,ab. OR (change or changing) adj (behavio?r or practice).ti,ab.) | 56489 |
| 3 | #1 AND #2 | 718 |
| 4 | limit 3 to yr="2015 -Current" | 246 |
